# Consequences of Workplace Ostracism: A Meta-Analytic Review

**DOI:** 10.3389/fpsyg.2021.641302

**Published:** 2021-08-02

**Authors:** Miaomiao Li, Xiaofeng Xu, Ho Kwong Kwan

**Affiliations:** ^1^Department of Business Administration, School of Economics and Management, Tongji University, Shanghai, China; ^2^Department of Organizational Behavior and Human Resource Management, China Europe International Business School (CEIBS), Shanghai, China

**Keywords:** workplace ostracism, meta-analysis, consequences, organization-based self-esteem, individualism-collectivism

## Abstract

Workplace ostracism, which is regarded as “social death,” is rampant in organizations and has attracted significant research attention. We extend the understanding of workplace ostracism by conducting a meta-analysis of studies of the relationships between workplace ostracism and its consequences. We also explore the moderating effects of national culture (i.e., collectivism vs. individualism) and the mediating effects of organization-based self-esteem (OBSE). The results of a meta-analysis of 95 independent samples (*N* = 26,767) reveal that exposure to workplace ostracism is significantly related to individuals’ attitudes, well-beings, and behaviors. Moreover, the effects of workplace ostracism on belongingness, job satisfaction, emotional exhaustion, organizational citizenship behavior (OCB) toward individuals (OCBI), organizational deviance, and interpersonal deviance are stronger in individualist contexts than in collectivist contexts. However, the relationships between workplace ostracism and organizational identification and OCB are stronger in collectivist contexts than in individualist contexts. Our meta-analytical structural equation modeling also provides evidence of the mediating effects of OBSE on the relationships between workplace ostracism and organizational commitment, job satisfaction, and job performance. The implications and limitations of our study and future research directions are also discussed.

## Introduction

Workplace ostracism, defined as “the extent to which an individual perceives that he or she is ignored or excluded by others” in the workplace (Ferris et al., 2008, p. 1348), can have significant consequences for organizations and individuals (Howard et al., 2020). The consequences of workplace ostracism for victims have been widely researched in the management literature [for reviews, see Mao et al. (2018), Williams (2007), and Wu et al. (2011)]. An individual who is ostracized by another party (e.g., colleagues or supervisors) in a dyadic relationship may experience injury, loss, or misfortune (Aquino and Lamertz, 2004). Whether intentional or unconscious, ostracism is a form of punishment, leading the ostracized victims to feel pain and need-threatened (Williams, 2009). From the victims’ perspective, workplace ostracism is associated with reduced organizational identification (Wu et al., 2016) and organizational commitment (Ferris et al., 2008) and increased psychological distress (Yaakobi and Williams, 2016), turnover intentions (Fiset et al., 2017), and deviant behavior in the workplace (Ferris et al., 2008).

To date, there has been one published meta-analytic review of workplace ostracism, except for Howard et al. (2020). Although that meta-analysis of workplace ostracism tests the antecedents and outcomes of ostracism, it only examines the bivariate relation, ignoring the boundary condition of cultural values and the mediation mechanisms. Our meta-analysis offers some extensions. Our review provides a more accurate evaluation of the consequences of workplace ostracism; for example, we note that studies have not examined the different dimensions of organizational citizenship behavior (OCB) and deviance, such as OCB toward the organization (OCBO) or toward individuals (OCBI) (Eatough et al., 2011), or interpersonal deviance or organizational deviance (Bennett and Robinson, 2000). We divide the identified consequences of workplace ostracism into three categories: attitudes, well-beings, and behaviors.

To explore the boundary condition of workplace ostracism bivariate relations, we examine the moderating effect of cultural differences. There have been no systematic meta-analyses of workplace ostracism in relation to contextual factors such as culture. Research suggests that cultural factors can enhance our understanding of the consequences of workplace ostracism (Mao et al., 2018). As one dimension of Hofstede’s (1980) influential study on cultural difference, individualism-collectivism emphasizes “the relationship between individual and the collectivity in a given society” (Bochner and Hesketh, 1994, p. 236). These two cultural values are strongly linked to an individual’s beliefs and behavior and can thus influence the relationships between workers (Bochner and Hesketh, 1994). Hence, this study focuses on the moderating effects of individualism-collectivism (Hofstede, 2001). Although Mao et al. (2018) examine the implications of cultural values in their qualitative review, they call for studies to specifically examine the moderating role of cultural values in the relationships between workplace ostracism and its outcomes. Thus, our meta-analysis provides the first quantitative review of the moderating effects of cultural values on the relationships between workplace ostracism and its consequences.

To investigate the mechanism of workplace ostracism, this study uses meta-analytic structural equation modeling (MASEM). Howard et al. (2020) fail to examine a number of important consequences of workplace ostracism, such as organization-based self-esteem (OBSE), although prior studies have shown that workplace ostracism is negatively related to OBSE (Ferris et al., 2008, 2015; Chung and Yang, 2017). Moreover, Howard et al. (2020) focus on the bivariate relationships between workplace ostracism and its outcomes but ignore the mediating mechanisms. To advance our knowledge of how victims suffer workplace ostracism, our study examines the mediating role of OBSE, which is defined as “one’s belief about his or her self-worth and competence as an organizational member” (Bowling et al., 2010, p. 601). According to the self-consistency motivational theory (Korman, 1976), workplace ostracism damages one’s self-evaluation, which may yield negative outcomes on affect and behaviors. However, there are no studies of the mediating effect of OBSE and its specific outcomes (e.g., organizational commitment, job satisfaction, and job performance). Thus, we examine OBSE because individuals are motivated to maintain their self-esteem (Korman, 1976), and workplace ostracism induces negative effects by damaging workers’ self-evaluations (Ferris et al., 2015).

Our meta-analytic study makes three main contributions to the literature. First, we review more studies of the consequences of workplace ostracism than Howard et al. (2020), including more published empirical studies, and find stronger evidence of the destructive effects of workplace ostracism. Second, by examining the moderating effects of individualism-collectivism on the relationships between workplace ostracism and its consequences, we enrich our knowledge of the importance of cultural factors on this bivariate relation. We thus also answer the calls to consider the effects of culture values on the consequences of workplace ostracism. Third, we construct a meta-analytical structural equation model (MASEM) of workplace ostracism to examine the mediating effects of OBSE on the relationships between workplace ostracism and its consequences. This approach enables us to integrate some of the outcomes of workplace ostracism.

## Theory and Hypotheses

### Overall Framework

**FIGURE 1:**
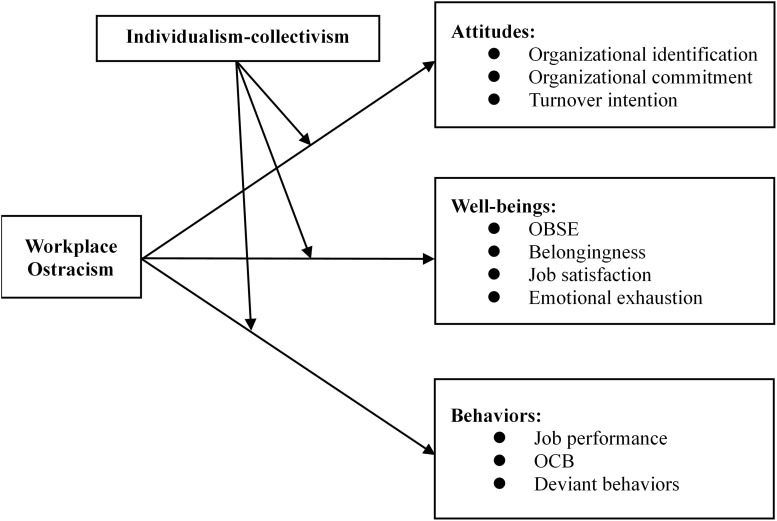
Theoretical framework for outcomes of workplace ostracism.

Consistent with previous meta-analyses of negative behaviors in the workplace (Greco et al., 2019; Howard et al., 2020), our meta-analysis combines three approaches. First, ostracism is a powerful threat to people’s need for belonging, self-esteem, shared understanding, and trust (Williams, 2007). When individuals feel ostracized, they may express different affective, attitudinal, and behavioral reactions (Zhang and Liao, 2015; Mao et al., 2018). Adopting the victims’ perspective (Aquino and Lamertz, 2004), we test the consequences of workplace ostracism by dividing all outcomes into three categories: attitudes, well-beings, and behaviors (see Figure 1). Second, ostracism does not lead to the same reactions across different cultures. To consider the specific consequences in a given culture and thus respond to the call to confirm that culture matters (Mao et al., 2018), we focus on individualism-collectivism as the moderator to examine how national culture influences the bivariate relation between workplace ostracism and its consequences. Third, we use our MASEM to examine the mediating effects of OBSE on the relationships between workplace ostracism and some of the consequences that we review. Specifically, we examine the mediating effect of OBSE on the relation between workplace ostracism and three main consequences: organizational commitment, job satisfaction, and job performance. Including all bivariate relations in the theoretical model is impossible due to “the absence of primary studies needed to complete a larger matrix of meta-analytically derived correlations between all possible pairs of variables included in structural model” (Lapierre et al., 2018, p. 390). Therefore, we consider three consequences that are relatively well studied constructs in the ostracism literature and are representative of the effect of workplace ostracism on attitudes, well-beings, and behaviors. According to the self-consistency motivational theory (Korman, 1976), OBSE motivates individuals who feel ostracized to pursue self-value, leading to higher organizational commitment, job satisfaction, and job performance.

### Workplace Ostracism and Attitudes

Workplace ostracism can cause the victim to experience pain and frustration, which may undermine his or her fundamental psychological needs and generate a sense of “social death” (Williams, 2007). When individuals perceive themselves as ostracized, they are likely to experience pain and have negative attitudes toward others and the organization (Mao et al., 2018). Workplace ostracism also signals separation from others and threatens the victim’s needs, undermining the victim’s sense of belonging (Williams, 2009). Moreover, workplace ostracism is often conducted in a silent and invisible manner, which undermines the victim’s sense of being valued as a member of the organization and reduces his or her organizational identification (Ferris et al., 2008; Wu et al., 2016). In addition, because workplace ostracism can deplete the victim’s personal resources, the victim may seek to protect his or her resources by reducing his or her organizational commitment or leaving the organization (Zheng et al., 2016). Thus, we propose the following hypothesis.

Hypothesis 1: Workplace ostracism is negatively related to positive attitudinal outcomes such as organizational identification (H1a) and organizational commitment (H1b) and positively related to negative employee attitudes such as turnover intentions (H1c).

### Workplace Ostracism and Well-Beings

Workplace ostracism can have a strong effect on an individual’s sense of well-being. Well-being, which can range from a negative condition (e.g., misery) to a positive condition (e.g., elation), reflects an individual’s psychological state, which is a broad category that includes emotional responses, domain satisfactions, and global judgments of life satisfaction (Diener et al., 1999). In the workplace, ostracism is characterized by omission of inaction to socially engage another and the lack of social engagement with others (Robinson et al., 2013). When individuals experience reduced social interaction, it can lead them to feel they are like dead to others in the workplace, which undermines the sense of self-value they gain from the organization and makes them doubt themselves (Williams, 2009). Omission of inaction by another organizational member when it is socially appropriate to do so can also lead individuals to blame themselves for being ostracized (Robinson et al., 2013). Because being ostracized leads the victims to perceive themselves as unwelcome to others or the organization (Ferris et al., 2008), workplace ostracism can damage OBSE.

According to belongingness theory (Baumeister and Leary, 1995), individuals strive to be accepted and to gain a sense of belonging. Through omission of inaction, workplace ostracism serves as negative feedback and thus damages the victim’s sense of belonging. Moreover, workplace ostracism can bring social pain and generate negative effects such as decreased job satisfaction (Ferris et al., 2008; Robinson et al., 2013). Workplace ostracism can also require the victim to exert more effort in dealing with interpersonal demands, which can make the individual “feel drained and overwhelmed by their work” (Wilk and Moynihan, 2005, p. 917) and emotionally exhausted. Thus, we propose the following hypothesis.

Hypothesis 2: Workplace ostracism is negatively related to elements of a positive sense of well-being such as OBSE (H2a), belongingness (H2b), and job satisfaction (H2c) and positively related to elements of a negative sense of well-being such as emotional exhaustion (H2d).

### Workplace Ostracism and Behaviors

Workplace ostracism can have either antisocial or prosocial behavioral implications (Mao et al., 2018). Although workplace ostracism can have beneficial consequences for individuals who regain acceptance (e.g., OCB), they still tend to be more socially susceptible and to have increased desires for conformity, compliance, and obedience (Carter-Sowell et al., 2008; Riva et al., 2014). Numerous studies have examined the detrimental consequences of workplace ostracism in relation to the victim’s psychological needs and behavior (Baumeister and Leary, 1995; Williams, 2007). Consistent with the “eye for an eye” mentality, workplace ostracism can trigger negative reciprocity, such that ostracism is linked to negative responses, including deviant behavior (Greco et al., 2019). Bennett and Robinson (2000) suggest that deviance can be directed toward either the organization (organizational deviance) or individuals (interpersonal deviance) based on qualitative (e.g., motives to commit crime) and quantitative distinctions (e.g., distinct clusters). Thus, ostracized individuals may engage in more interpersonal deviance, such as spreading rumors or being physically violent toward colleagues, when their sense of belonging has been undermined (Baumeister and Leary, 1995). Ostracized individuals may also engage in organizational deviance, such as by sabotaging equipment and littering the workplace environment, when they do not receive support from their organizations and feel that their identity in the workplace is threatened (Ferris et al., 2009). Moreover, the reciprocity norms of good-with-good and bad-with-bad suggest that ostracized individuals may repay the organization with less positive behavior, such as by reducing their job performance and OCB. Specifically, ostracized individuals are likely to reduce their OCBO if they perceive a loss of resources as a result of experiencing omission from the organization, whereas ostracized individuals are likely to reduce their OCBI if they fail to build and maintain good relationships with their coworkers (Eatough et al., 2011). Thus, we propose the following hypothesis.

Hypothesis 3: Workplace ostracism is positively related to negative behaviors such as organizational deviance (H3a) and interpersonal deviance (H3b) and is negatively related to individual positive behaviors such as job performance (H3c), OCB (H3d), OCBO (H3e), and OCBI (H3f).

### Moderating Effects of Individualism-Collectivism

Individualism refers to the pursuit of individual interests (Waterman, 1984), whereas collectivism signifies a society-centered orientation and the pursuit of common interests, such as the “sharing of material benefits and non-material resources” (Hui and Triandis, 1986, p. 225). We propose that the relationships between workplace ostracism and its consequences are stronger in individualist than in collectivist cultures for two reasons. First, individuals with a strong sense of collectivism cherish their connections with the organization and interpersonal relationships, and are more tolerant of workplace ostracism even if they feel uncomfortable about being excluded. In contrast, individuals with a strong sense of individualism care more about themselves than their relationships, and they are extremely sensitive to others’ attitudes and how others treat them (Rockstuhl et al., 2012). Second, individuals with a strong sense of collectivism seek to maintain social harmony and identify with their organizations for moral reasons, whereas individuals with a strong sense of individualism are motivated by personal calculation and the utilitarian pursuit of their self-interests (Bochner and Hesketh, 1994). Hence, collectivist individuals tend to exercise self-restraint and to be more motivated to mitigate the negative effects of workplace ostracism on their attitudes, sense of well-beings, and behaviors (Yaakobi and Williams, 2016). In contrast, people in individualist cultures pursue equal treatment, and their personal relationships are more influenced by others’ reactions (Rockstuhl et al., 2012). Consequently, we propose the following hypotheses.

Hypothesis 4: The relationship between workplace ostracism and the victim’s attitudes is stronger in individualist cultures than in collectivist cultures.

Hypothesis 5: The relationship between workplace ostracism and the victim’s well-beings is stronger in individualist cultures than in collectivist cultures.

Hypothesis 6: The relationship between workplace ostracism and the victim’s behaviors is stronger in individualist cultures than collectivist cultures.

### OBSE as a Mediator of the Relationship Between Workplace Ostracism and Outcomes

As a form of self-evaluation shaped by experience, OBSE has strong effects on individuals’ motivation, attitudes, and behaviors (Pierce et al., 1989). In their meta-analysis of OBSE, Bowling et al. (2010) report the relationships between OBSE and organizational commitment (*r* = 0.55), job satisfaction (*r* = 0.57), and in-role performance (*r* = 0.34). Workplace ostracism, as a “real” measure of interpersonal evaluation, is an expression of others’ thoughts about the victim through their behavior and can hurt the victim by destroying his or her sense of competence and need-satisfaction (Korman, 1976). Moreover, the low levels of OBSE observed following workplace ostracism lead individuals to evaluate themselves as “useless failures” (Ferris et al., 2015), inducing them to exhibit less organizational commitment, lower job satisfaction, and poorer job performance. Thus, we propose the following hypothesis.

Hypothesis 7: OBSE mediates the relationships between workplace ostracism and organizational commitment (7a), job satisfaction (7b), and job performance (7c).

## Materials and Methods

### Literature Search and Inclusion Criteria

**FIGURE 2:**
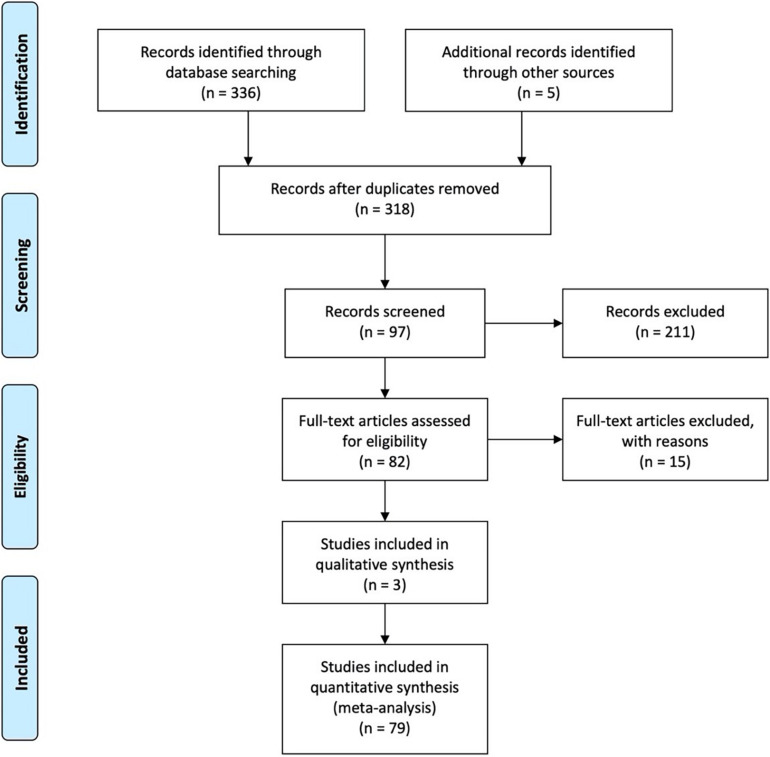
The literature research procedure.

To study the relationships between workplace ostracism and the outcome variables in our meta-analysis, we first conducted a computerized search of several databases, including the Web of Science, PsycINFO, EBSCO, ProQuest Dissertations, and China National Knowledge Internet (CNKI), using “ostracism,” “exclusion,” “rejection,” and “isolation” as keywords. We obtained 132 articles from Web of Science, 64 articles from PsycINFO, 35 articles from EBSCO, eight articles from ProQuest Dissertations, and 97 articles from CNKI. In addition, we manually checked the reference lists of the reviews by Howard et al. (2020) and Mao et al. (2018) to ensure that we did not overlook any relevant study. To find unpublished studies, we also searched the Academy of Management 2009–2019 Annual Meeting and Society for Industrial and Organizational Psychology 2016–2019 Annual Conference programs and called for unpublished or in-press manuscripts *via* e-mail. Five scholars responded to the emails and offered their manuscripts. The literature research procedure is shown as Figure 2.

After these thorough searches, our inclusion criteria were as follows. First, the timeline was from 2008 to 2019. 2008 was chosen because the first scale for measuring workplace ostracism was developed and published in that year (Ferris et al., 2008). Second, we included the 10-item scale developed by Ferris et al. (2008) to measure workplace ostracism as an inclusion criterion. Third, each study needed to provide the statistical information required to compute an effect size, such as the product-moment correlation coefficient (r), Cronbach’s alpha (α), and sample size. Fourth, to enable us to examine the moderating role of collectivism-individualism, using samples from the countries in primary studies as a proxy of cultural value is the analytical technique in the meta-analysis procedure. Samples from the countries in primary studies are represent to correspond cultural values of individualism or collectivism based on data from the website^1^. After excluding 33 overlapping articles and 229 ineligible articles, our sample consisted of 79 sources—38 sources in Chinese and 41 in English—discussing 95 studies. All primary empirical studies in meta-analysis are shown in the references marked with an (^∗^) asterisk. The sample included one unpublished manuscript. To ensure that the data were coded correctly, two researchers independently coded and recorded the effect sizes and other necessary information about the focal relationships. The researchers checked their coding together and found that the consistency was over 95%. All of the authors discussed any discrepancies in the coding until consensus was reached.

### Analyses

We used Hunter and Schmidt’s (2004) meta-analytic procedure to compute the results and calculated the product-moment correlation coefficient (*r*) for each study to show the effect size. We also corrected each correlation for unreliability in measurement by reporting the Cronbach’s alphas (α) of each study. We recorded the key indexes, such as the independent effect size (*k*), cumulative sample size (*N*), sample size weighted mean observed correlation ($\bar{r}$), mean true score correlation (ρ), standard deviation of the observed correlations (SD_$\bar{r}$_ standard deviation of the true score correlation (SD_ρ_), 95% confidence interval, 80% credibility interval, and percentage of variation in the observed correlations attributable to sampling error and other factors (% acc). A 95% confidence interval excluding zero indicated that the corrected correlation was statistically significant. Moreover, a sufficiently large 80% credibility interval or an interval that included zero provided information about the possibility of moderators (Whitener, 1990; Yu et al., 2016). Furthermore, a percentage of variation less than 75% indicated the possibility of moderators (Hunter and Schmidt, 2004).

To test the moderating effects of collectivism-individualism, we recoded the sample locations as either Asia (including China, Korea, and Pakistan) or the Occident (including Netherlands, Canada, Cyprus, Spain, and the United States) to proxy for collectivism and individualism. Asia and the Occident represent typical regions with collectivist and individualist cultural orientations, respectively (Triandis and Gelfand, 1998). We calculated the separate meta-analytic effects for each relationship for each region. Using these results (i.e., the mean true score correlation and cumulative sample size of each subgroup), we also calculated the Q statistic between-group homogeneity coefficient, which we used to assess whether the effects were homogeneous or heterogeneous between groups. A significant Q statistic indicates heterogeneity, and the moderators can explain the source of the heterogeneity. Lastly, we calculated a measure of potential heterogeneity (*Q*), the *p*-value for the Q statistic, the standard deviation of the true effect size (*T*), and a measure of the proportion of dispersion that can be attributed to real differences in the effect sizes as opposed to within-study error (*I*^2^).

**FIGURE 3:**
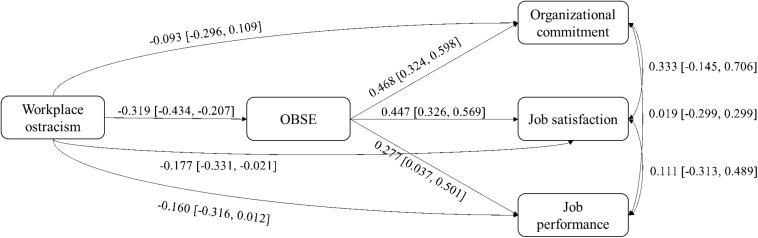
Results of full-information meta-analytic structural equation modeling test.

We also constructed a MASEM to test the theoretical (path) model presented in Figure 3. To evaluate the path model by using online software^2^, we used the full-information MASEM method to account for the heterogeneity of the effect sizes (Yu et al., 2016). During the input procedure, we constructed and used the matrixes of ρ and SD_ρ_. The correlations between workplace ostracism and its outcomes then provided the input matrix cell values. Additional values were collected from several meta-analyses, including Bowling et al. (2010), Judge et al. (2001), Mathieu and Zajac (1990), and Riketta (2002). Table 1 provides a matrix of the input values. We constructed the two required matrices, and the SEM was estimated for each bootstrapped matrix (Yu et al., 2016). This analysis generated the path model parameter estimates and SEM fit index values.

**TABLE 1:** Input matrices for full-information meta-analysis structure equation modeling.

Variable	Workplace ostracism				OBSE				Organizational commitment				Job satisfaction			
				
	ρ	SD_ρ_	*k*	*N*	ρ	SD_ρ_	*k*	*N*	ρ	SD_ρ_	*k*	*N*	ρ	SD_ρ_	*k*	*N*
Workplace ostracism	—															
OBSE	−0.337	0.093	11	2421	—											
Organizational commitment	−0.281	0.167	8	3335	0.55	0.10	24	8403	—							
Job satisfaction	−0.377	0.132	12	2363	0.57	0.09	34	10362	0.533	0.241	43	15531	—			
Job performance	−0.267	0.100	17	4261	0.34	0.17	12	2020	0.198	0.108	111	26344	0.30	0.21	312	54471

**k*, number of independent samples cumulated; *N*, cumulative sample size; ρ, mean true score correlation; SD_ρ_, standard deviation of the true score correlation.*

To examine the publication bias analyses of workplace ostracism consequences, we used R packages^3^, such as metafor, dmeta, and weightr. We reported fail-safe k, Egger’s test, random effects trim-and-fill method, and weight-function model analysis. Estimation of publication bias should meet the following requirements: the fail-safe *k* should be sufficiently large (*N* > 50); Egger’s test should be non-significant (*p* < 0.05); a random-effects trim-and-fill method with the missing mean (*k* > 3) suggests that publication bias is present; and the weight-function model with specified *p*-values intervals (*p* < 0.05 and *p* > 0.05) should be non-significant (Vevea and Hedges, 1995; Vevea and Woods, 2005; Vevea and Coburn, 2015).

## Results

### Main Effect

As shown in Table 2, workplace ostracism is significantly and negatively related to organizational identification (ρ = −0.372), organizational commitment (ρ = −0.281), OBSE (ρ = −0.337), belongingness (ρ = −0.293), job satisfaction (ρ = −0.377), job performance (ρ = −0.267), OCB (ρ = −0.256), OCBO (ρ = −0.282), and OCBI (ρ = −0.256), and positively related to turnover intentions (ρ = 0.303), emotional exhaustion (ρ = 0.428), organizational deviance (ρ = 0.610), and interpersonal deviance (ρ = 0.570). All of the 95% confidence intervals exclude 0. Thus, Hypotheses 1, 2, and 3 are supported. In addition, the results of the 80% credibility interval and % acc indicate that except for OCBO, the relationships between workplace ostracism and its consequences may involve moderators.

**TABLE 2:** Meta-analysis results of consequences of workplace ostracism.

Variable	*k*	*N*	$\bar{r}$	SD_$\bar{r}$_	ρ	SD_ρ_	SDr_*c*_	95% confidence interval	80% credibility interval	% acc
**Attitudes**										
Organizational identification	13	3294	–0.327	0.190	–0.372	0.219	0.229	(−0.495, −0.249)	(−0.653, −0.091)	8
Organizational commitment	8	3335	–0.243	0.126	–0.281	0.153	0.162	(−0.392, −0.170)	(−0.477, −0.085)	11
Turnover intentions	19	5061	0.265	0.073	0.303	0.090	0.111	(0.255, 0.351)	(0.188, 0.417)	35
**Well-beings**										
OBSE	11	2421	–0.300	0.081	–0.337	0.093	0.116	(−0.403, −0.271)	(−0.456, −0.218)	36
Belongingness	6	1721	–0.238	0.221	–0.293	0.257	0.265	(−0.503, −0.083)	(−0.622, 0.036)	6
Job satisfaction	12	2363	–0.333	0.112	–0.377	0.124	0.143	(−0.455, −0.298)	(−0.535, −0.219)	25
Emotional exhaustion	8	1858	0.363	0.092	0.428	0.108	0.127	(0.343, 0.512)	(0.290, 0.565)	28
**Behaviors**										
Job performance	17	4261	–0.243	0.083	–0.267	0.100	0.120	(−0.322, −0.211)	(−0.395, −0.139)	30
OCB	12	2522	–0.219	0.148	–0.256	0.172	0.188	(−0.360, −0.152)	(−0.476, −0.036)	17
OCBO	8	1875	–0.249	0.000	–0.282	0.000	0.067	(−0.324, −0.239)	(−0.282, −0.282)	108
OCBI	10	2430	–0.229	0.041	–0.256	0.047	0.083	(−0.303, −0.208)	(−0.316, −0.196)	68
Organizational deviance	23	8676	0.555	0.213	0.610	0.222	0.225	(0.518, 0.701)	(0.326, 0.893)	3
Interpersonal deviance	21	6583	0.513	0.203	0.570	0.218	0.223	(0.475, 0.665)	(0.291, 0.849)	4

**k*, number of independent samples cumulated; *N*, cumulative sample size; % acc, percentage of variation in the observed correlations attributable to sampling error and other artifacts; r̄, sample size weighted mean observed correlation; ρ, mean true score correlation; SDr̄, standard deviation of the observed correlations; SDrc, observed standard deviation of the corrected correlations; SDρ, standard deviation of the true score correlation.*

### Moderating Effects

As shown in Table 3, the between-group Q-statistics for all of the relationships except for those between workplace ostracism and organizational commitment, turnover intentions, OBSE, and job performance are significant. In terms of attitudinal outcomes, the results indicate that the relationship between workplace ostracism and organizational identification is significantly different (*Q* = 6.962, *p* < 0.01) and stronger in the collectivist culture subgroup (ρ*_*c*_* = −0.385) than in the individualist culture subgroup (ρ*_*i*_* = −0.230). The relationships between workplace ostracism and organizational commitment (*Q* = 3.115, *ns*) and turnover intentions (*Q* = 0.521, *ns*) are not significantly different. Thus, Hypothesis 4 is not supported.

**TABLE 3:** The moderating effect of individualism-collectivism on workplace ostracism-consequence relationships.

Variable	Moderator subgroup	*k*	*N*	$\bar{r}$	SD_$\bar{r}$_	ρ	SD_ρ_	95% confidence interval	80% credibility interval	% acc	*Q*	*p*	I^2^	*T*
**Attitudes**														
Organizational identification	Collectivism	11	3035	–0.337	0.196	–0.385	0.226	(−0.523, −0.248)	(−0.675, −0.096)	7	6.962	0.008	85.64	0.112
	Individualism	2	259	–0.209	0.000	–0.230	0.000	(−0.347, −0.113)	(−0.230, −0.230)	559				
Organizational commitment	Collectivism	6	1379	–0.271	0.121	–0.315	0.154	(−0.447, −0.182)	(−0.511, −0.118)	18	3.115	0.078	67.90	0.036
	Individualism	2	1956	–0.224	0.126	–0.258	0.149	(−0.468, −0.047)	(0.448, −0.067)	5				
Turnover intentions	Collectivism	15	3895	0.269	0.082	0.307	0.103	(0.248, 0.367)	(0.177, 0.439)	30	0.521	0.470	–91.81	0
	Individualism	4	1166	0.250	0.015	0.286	0.000	(0.233, 0.340)	(0.286, 0.286)	112				
**Well-beings**														
OBSE	Collectivism	9	2265	–0.306	0.072	–0.341	0.087	(−0.410, −0.273)	(−0.453, −0.230)	35	0.929	0.335	–7.64	0
	Individualism	2	156	–0.213	0.135	–0.268	0.141	(−0.515, −0.022)	(0.448, −0.088)	46				
Belongingness	Collectivism	3	1215	–0.155	0.133	–0.189	0.165	(−0.384, 0.005)	(−0.400, 0.022)	12	47.628	0.000	97.90	0.256
	Individualism	3	506	–0.438	0.262	–0.506	0.282	(−0.832, −0.179)	(−0.867, −0.145)	6				
Job satisfaction	Collectivism	3	960	–0.269	0.129	–0.304	0.137	(−0.470, −0.137)	(−0.479, −0.128)	15	11.910	0.001	91.60	0.098
	Individualism	9	1403	–0.376	0.072	–0.429	0.080	(−0.498, −0.360)	(−0.531, −0.327)	49				
Emotional exhaustion	Collectivism	5	1059	0.328	0.060	0.380	0.076	(0.295, 0.466)	(0.283, 0.478)	47	9.059	0.003	88.96	0.094
	Individualism	3	799	0.409	0.106	0.494	0.111	(0.356, 0.632)	(0.352, 0.636)	23				
**Behaviors**														
Job performance	Collectivism	14	3920	–0.239	0.078	–0.262	0.095	(−0.319, −0.204)	(−0.383, −0.140)	30	1.578	0.209	36.62	0.030
	Individualism	3	341	–0.287	0.125	–0.327	0.137	(−0.510, −0.143)	(−0.502, −0.151)	34				
OCB	Collectivism	6	1653	–0.303	0.077	–0.350	0.085	(−0.431, −0.269)	(−0.459, −0.241)	36	51.576	0.000	98.06	0.211
	Individualism	6	869	–0.060	0.116	–0.064	0.142	(−0.195, 0.068)	(−0.245, 0.118)	33				
OCBI	Collectivism	7	1945	–0.211	0.014	–0.235	0.020	(−0.280, −0.190)	(−0.260, −0.210)	92	4.777	0.029	79.07	0.070
	Individualism	3	485	–0.304	0.027	–0.337	0.037	(−0.429, −0.246)	(−0.385, −0.290)	82				
Organizational deviance	Collectivism	16	4420	0.405	0.170	0.452	0.201	(0.350, 0.554)	(0.195, 0.710)	7	525.845	0.000	99.81	0.348
	Individualism	7	4256	0.712	0.118	0.753	0.115	(0.666, 0.839)	(0.606, 0.900)	3				
Interpersonal deviance	Collectivism	13	3757	0.422	0.206	0.480	0.251	(0.341, 0.619)	(0.159, 0.801)	5	131.749	0.000	99.24	0.201
	Individualism	8	2826	0.634	0.120	0.669	0.109	(0.590, 0.747)	(0.529, 0.808)	9				

*Collectivism includes China, Korea, Pakistani; Individualism includes Netherlands, Canada, America, Cyprus, Spain. *k*, number of independent samples cumulated; *N*, cumulative sample size; % acc, percentage of variation in the observed correlations attributable to sampling error and other artifacts; $\bar{r}$, sample size weighted mean observed correlation; ρ, mean true score correlation; SD_$\bar{r}$_, standard deviation of the observed correlations; SDr_*c*_, observed standard deviation of the corrected correlations; SD_ρ_, standard deviation of the true score correlation; Q, potential heterogeneity; *p*, *p*-value for the Q statistic; *T*, the standard deviation of true effect sizes; I^2^, the proportion of dispersion that can be attributed to real differences in effect sizes as opposed to within-study error.*

Regarding the well-being outcomes, the relationships between workplace ostracism and belongingness (ρ*_*i*_* = −0.506 vs. ρ*_*c*_* = −0.189; *Q* = 47.628, *p* < 0.001), job satisfaction (ρ*_*i*_* = −0.429 vs. ρ*_*c*_* = −0.304; *Q* = 11.910, *p* < 0.01), and emotional exhaustion (ρ*_*i*_* = 0.494 vs. ρ*_*c*_* = 0.380; *Q* = 9.059, *p* < 0.01) are significantly different in the two subgroups and are stronger in the individualist culture subgroup than in the collectivist culture subgroup. However, the relationship between workplace ostracism and OBSE (*Q* = 0.929, *ns*) is not significantly different. Thus, Hypothesis 5 is partially supported.

Finally, in terms of the behavioral outcomes, the relationships between workplace ostracism and OCBI (ρ*_*i*_* = −0.337 vs. ρ*_*c*_* = −0.235; *Q* = 4.777, *p* < 0.05 s), organizational deviance (ρ*_*i*_* = 0.753 vs. ρ*_*c*_* = 0.452; *Q* = 525.845, *p* < 0.001), and interpersonal deviance (ρ*_*i*_* = 0.669 vs. ρ*_*c*_* = 0.480; *Q* = 131.749, *p* < 0.001) are significantly different and stronger in the individualist culture subgroup than in the collectivist culture subgroup. The relationship between workplace ostracism and OCB (ρ*_*i*_* = −0.064 vs. ρ*_*c*_* = −0.350; *Q* = 51.576, *p* < 0.001) is significantly different but stronger in the collectivist culture subgroup than in the individualist culture subgroup. However, the relationship between workplace ostracism and job performance (*Q* = 1.578, *ns*) is not significantly different in the collectivist and individualist culture subgroups. Thus, Hypothesis 6 is partially supported.

### MASEM Analysis of Mediating Effects

The MASEM results indicate that the hypothesized model in which OBSE mediates the relationships between workplace ostracism and employees’ organizational commitment, job satisfaction, and job performance is largely generalizable across populations. Numerous fit indices were used to calculate the generalizability of the model. Specifically, the standardized root mean square residual value is smaller than 0.10 in 81.2% of the 500 bootstrapped iterations, and the comparative fit index value is 0.80 or above in 86% of the 500 bootstrapped iterations. The path coefficients and their 80% credibility intervals are shown in Figure 2. The path coefficient linking workplace ostracism with OBSE has a mean value of −0.319 [80% CV (−0.434, −0.207)]. The path coefficients linking OBSE to organizational commitment, job satisfaction, and job performance have mean values of 0.468 [80% CV (0.324, 0.598)], 0.447 [80% CV (0.326, 0.569)], and 0.277 [80% CV (0.037, 0.501)], respectively. The CV widths of all of the path coefficients are sufficiently large and credible. Thus, Hypothesis 7 is supported.

As shown in Table 4, estimation of publication bias should meet the requirements. The publication bias is concerning, our inspection indicated that it is not a big issue for our findings.

**TABLE 4:** Publication bias analyses of workplace ostracism consequences.

Variable	*R*	I^2^	*k*	Fail Safe k	Egger’s Test *t*	Implied missing		Weight function model	
		
						Left of mean	Right of mean	0.05 < *p* < 1	LR X^2^
**Attitudes**									
Organizational identification	–0.35	92.83	13	1903	0.37	0	0	2541.97	0.80
Organizational commitment	–0.28	81.43	8	582	–0.20	0	0	994.54	0.02
Turnover intentions	0.26	73.50	22	2641	1.48	3	0	0.01	3.12
**Well-beings**									
OBSE	–0.30	67.09	11	836	0.97	0	0	65.21	0.004
Belongingness	–0.28	94.68	6	245	–0.36	0	0	44994.56	1.27
Job satisfaction	–0.34	74.09	12	1103	0.41	0	0	149.85	0.01
Emotional exhaustion	0.39	78.26	8	789	0.13	0	0	0.01	0.46
**Behaviors**									
Job performance	–0.28	72.47	17	1561	–0.23	3	0	74.24	0.03
OCB	–0.26	73.69	12	602	0.41	1	0	84.68^a^	0.07
OCBO	–0.23	20.89	8	353	–1.31	0	2	149.87	0
**OCBI**	–0.24	37.84	10	475	–0.43	0	0	0.01	0.04
Organizational deviance	0.59	97.09	23	20351	–4.70	0	5	0.01	7.57**
Interpersonal deviance	0.66	97.27	21	14416	–2.07	0	6	0.34	0.63

*^*a*^At least one of the *p*-value intervals contains no effect sizes, re-specifying the outpoints of 0.04 < *p* < 1.*

## Discussion

Adopting the victims’ perspective, our meta-analytical review tests the bivariate relations of workplace ostracism on attitudes, well-being, and behaviors using a version of the measure developed by Ferris et al. (2008). Our findings largely support the theoretical model shown in Figure 1 based on our review of empirical studies. Specifically, we find that 13 pairs of bivariate relations are significant. This meta-analysis clarifies the moderating effect of individualism-collectivism, as the findings are partially supportive of these boundary conditions. We further synthesize the MASEM to highlight the mediating effects of OBSE on the relationship between workplace ostracism and some specific consequences (i.e., organizational commitment, job satisfaction, and job performance).

### Theoretical Implications

Our meta-analysis of the literature has three main theoretical implications. First, in our tests of the psychometric corrections, we systemically evaluate the frameworks on workplace ostracism and its consequences, including more studies (*k* = 95 studies of the consequences of workplace ostracism) than Howard et al. (2020) (*k* = 93 studies of the antecedents and consequences of workplace ostracism). We also use a fine-grained approach to test OCB and deviance targeted at organizations or individuals, offering evidence that workplace ostracism significantly affects organizations and individuals.

Second, we test the moderating effects of individualism-collectivism on the relationships between workplace ostracism and its outcomes, and thus respond to the call of Mao et al. (2018) to examine workplace ostracism across different cultural contexts. Our findings suggest that national cultures play an important role in determining the boundary conditions of workplace ostracism and its consequences. The individualism-collectivism divide is one of the most salient factors affecting the workplace environments in which individuals are embedded (Wong and Cheng, 2020). We generate three findings that show the complex cultural values of individualism-collectivism. First, consistent with our hypotheses, we find that the relationships between workplace ostracism and belongingness, job satisfaction, emotional exhaustion, OCBI, organizational deviance, and interpersonal deviance are stronger in individualist contexts than in collectivist contexts. On this basis, we propose that people with a strong sense of individualism are more sensitive to how others treat them (Rockstuhl et al., 2012). Second, contrary to our hypothesis, we find that the relationships between workplace ostracism and organizational identification and OCB are stronger for individuals with a strong sense of collectivism. Drawing on social identity theory, Wu et al. (2016) suggest that collectivism may strengthen the relationship between workplace ostracism and organizational identification, but they do not find a significant relationship. Our meta-analyses show that collectivism is indeed a moderator. This finding suggests that scholars should consider the outcome variables when examining the moderating role of culture (Lian et al., 2012). Third, our findings do not support the moderating effects of individualism-collectivism on organizational commitment, turnover intentions, OBSE, or job performance. That is, the correlations between workplace ostracism and these four variables are not significantly different between individualist and collectivist cultures. However, individualism-collectivism is represented by regional divisions and is not directly measured as a moderator in our analyses. This potential measurement error may have contributed to the finding that the hypothesized moderating effect is insignificant.

Third, we offer important empirical evidence of the mediating effects of OBSE on the relationships between workplace ostracism and organizational commitment, job satisfaction, and job performance using MASEM, demonstrating its superiority for theoretical models and analytical techniques. Meta-analytic research has shed light on the mediating effects of OBSE on self-esteem and work-related consequences (Bowling et al., 2010). Our research extends the predictors of OBSE by regarding workplace ostracism as an antecedent and adds to the mistreatment and OBSE literature by examining samples from heterogeneous populations. Our meta-analysis also improves the methodology that Howard et al. (2020) use to test the mediating effects of OBSE. Consequently, we have enriched the ostracism research by positioning OBSE as a mediator in our model. Our findings explain how workplace ostracism has destructive effects on victims’ job attitudes and performance. As OBSE provides a theoretical foundation for explaining how mistreatment affects behavior (Ferris et al., 2015), our findings could inspire researchers to focus on the mediating role of OBSE in the mistreatment literature.

### Practical Implications

Workplace ostracism brings pain and hurt to individuals (Williams, 2009). Thus, we must work to reduce the negative effects of workplace ostracism because of its undesirable consequences in relation to individuals’ attitudes, well-beings, and behaviors. Organizations should seek to establish and maintain a friendly atmosphere that offers a sense of belonging to employees, minimize workplace ostracism by creating a zero-tolerance culture, and provide training programs on how to avoid ostracism (Wu et al., 2012). Most importantly, organizations should pay more attention to OBSE and change the work environment when OBSE mediates the relationship between workplace ostracism and its consequences (Bowling et al., 2010). In addition, individuals should increase their awareness of workplace ostracism to prevent themselves from being ostracized. If workplace ostracism has badly affected an individual’s sense of well-being, they should seek help from the right person (e.g., other warm-hearted supervisors or colleagues) or the organizational department (Kwan et al., 2018).

### Limitations

The quality of the primary studies can affect the quality of a meta-analysis. Our study has two noteworthy limitations. First, our meta-analytical model could not test for causality, as it examines cross-sectional data from the sample studies. Cross-sectional data tend to have measurement bias, and strong tests require multiple sources and manipulation of the variables (Bono and McNamara, 2011). For example, a longitudinal design might offer a clear explanation of whether workplace ostracism precedes belongingness, whether belongingness precedes workplace ostracism, or whether they reciprocally influence each other. Second, the small number of cross-cultural samples included in the meta-analysis (*k* = 2 studies) may have led to organizational identification, organizational commitment, OBSE, and OCBO being falsely associated with individualism (Hunter and Schmidt, 2004). Third, the meta-analysis only considers English and Chinese publications, and does not include publications in other languages. Future meta-analysis of workplace ostracism may broaden the sample of publications to include more languages (e.g., Spanish, French). Finally, although we focus on the well-established dimension of individualism-collectivism, the Hofstede’s cultural dimensions may not account for some observed differences as a result of the cultural dimension of individualism, such as GDP, political ideology, economic conditions and so on. Using the national-level Hofstede’s cultural values may capture less variance than individual-level cultural values, which might trigger questions of accuracy. Thus, we believe that our findings of moderating effect of individualism-collectivism may be conservative. Future research could further explore moderating effects of Hofstede’s cultural dimensions by using a more specific cultural values perspective.

### Future Research Directions

In addition to assessing the causality and broadening the sample size, out study points to several directions for future research. First, the consequences of workplace ostracism are not limited to the workplace, as workplace ostracism can have spillover effects on the family (Liu et al., 2013). However, the limited samples on work-family interface variables means that we could not examine this factor in our meta-analysis of workplace ostracism. It is often difficult to segment the work and family domains, which suggests that a new direction is needed to explore the effects of workplace ostracism on the family. We encourage future researchers to conduct meta-analytic research on how workplace ostracism impacts the work-family interface when sufficient studies become available.

Second, the perpetrator and the victim may have different views of workplace ostracism (Yang and Treadway, 2018). In this study, we focus on the victims’ perceptions of workplace ostracism. We call for future meta-analyses to consider the sources of the ratings (i.e., by self or by other) of perceived ostracism and their potential moderating effects on the relationships between workplace ostracism and its consequences.

Third, future research should consider other moderators, such as personality. For example, research has shown that a proactive personality moderates the relationship between workplace ostracism and counterproductive work behavior (Zhao et al., 2013). Thus, the moderating effects of personality should be tested when the sample sizes of the studies meet the standards for meta-analysis.

Finally, we encourage future researchers to test other mediators of the relationships between workplace ostracism and its outcomes such as emotions (e.g., anger, anxiety), as these variables may help to explain the mediating mechanism between workplace ostracism and its outcomes (Ferris et al., 2016).

## Conclusion

Using an adapted version of the measure developed by Ferris et al. (2008), we construct a meta-analytical model to test the effects of workplace ostracism on the attitudes, well-beings, and behaviors of the victim. We also examine the moderating effects of collectivism-individualism on the relationships between workplace ostracism and its consequences. In addition, we use MASEM to test the mediating effects of OBSE on the relationships between workplace ostracism and its outcomes. We hope that our meta-analysis provides clear directions for future research on workplace ostracism and will encourage more researchers to examine workplace ostracism.

## Data Availability Statement

The raw data supporting the conclusions of this article will be made available by the authors, without undue reservation.

## Author Contributions

ML and HK were responsible for idea generation. ML and XX conducted material preparation, data collection, and analysis. ML wrote the first draft. HK and XX revised the manuscript. All authors commented on previous versions of the manuscript and read and approved the final manuscript.

## Conflict of Interest

The authors declare that the research was conducted in the absence of any commercial or financial relationships that could be construed as a potential conflict of interest.

## Publisher’s Note

All claims expressed in this article are solely those of the authors and do not necessarily represent those of their affiliated organizations, or those of the publisher, the editors and the reviewers. Any product that may be evaluated in this article, or claim that may be made by its manufacturer, is not guaranteed or endorsed by the publisher.

## Footnotes

1
https://www.hofstede-insights.com/country-comparison/

2
https://mgmt.shinyapps.io/masem/

3
https://bookdown.org/MathiasHarrer/Doing_Meta_Analysis_in_R/
