# Supplementary material for: Consequences of Workplace Ostracism: A Meta-Analytic Review
Source: Front Psychol. 2021 Aug 2;12:641302. doi: 10.3389/fpsyg.2021.641302 (PMC8365139; doi:10.3389/fpsyg.2021.641302)
Supplement: Supplementary file 1 [file Data_Sheet_1.ZIP › Supplementary Material Presentation./MASEM Syntax on the website.docx]

MASEM Syntax on the website

OBSE ~ WO

OC ~ OBSE

JS ~ OBSE

JP ~ OBSE

OC ~ WO

JS ~ WO

JP ~ WO

OC ~~JS

OC ~~ JP

JS ~~ JP
