# Supplementary material for: Consequences of Workplace Ostracism: A Meta-Analytic Review
Source: Front Psychol. 2021 Aug 2;12:641302. doi: 10.3389/fpsyg.2021.641302 (PMC8365139; doi:10.3389/fpsyg.2021.641302)
Supplement: Supplementary file 1 [file Data_Sheet_1.ZIP › Supplementary Material Presentation./Supplemental material A-Publication Bias Analyses.docx]

**Supplemental Material –** **Publication Bias Analyses**

We provide supplemental materials in the table as below to present publication bias analyses of workplace ostracism consequences by using packages (i.e., metafor, dmeta, and weightr) in R (https://bookdown.org/MathiasHarrer/Doing_Meta_Analysis_in_R/). Supplemental Table A presents the results of fail-safe k, Egger’s test, random effects trim-and-fill method, and weight-function model analysis. Estimation of publication bias should meet the following requirements: the fail-safe k should be sufficiently large (N > 50); Egger’s test should be nonsignificant (p < .05); a random-effects trim-and-fill method with the missing mean (k > 3) suggests that publication bias is present; and the weight-function model with specified p-values intervals (p < .05 and p > .05) should be nonsignificant (Vevea & Coburn, 2015; Vevea & Hedges, 1995; Vevea & Woods, 2005). Although the publication bias is concerning, our inspection indicated that it is not a big issue for our findings.

***References***

Vevea, J. L., & Coburn, K. M. (2015). Maximum-likelihood methods for meta-analysis: A tutorial using R. *Group Processes & Intergroup Relations*, 18(3), 329-347. doi:10.1177/1368430214558311

Vevea, J. L., & Hedges, L. V. (1995). A general linear-model for estimating effect size in the presence of publication bias. *Psychometrika*, 60(3), 419-435. doi:10.1007/Bf02294384

Vevea, J. L., & Woods, C. M. (2005). Publication bias in research synthesis: Sensitivity analysis using a priori weight functions. *Psychological Methods*, 10(4), 428-443. doi:10.1037/1082-989x.10.4.428

Viechtbauer, W., & Cheung, M. W. (2010). Outlier and influence diagnostics for meta-analysis. *Research Synthesis Methods*, 1(2), 112-125. doi:10.1002/jrsm.11

Publication Bias Analyses of Workplace Ostracism Consequences

| **Variable** | *r* | I^2^ | k | Fail Safe k | Egger’s Test t | Implied Missing | | Weight function model | |
| --- | --- | --- | --- | --- | --- | --- | --- | --- | --- |
|  |  |  |  |  |  | Left of Mean | Right of Mean | 0.05<p<1 | LR X^2^ |
| **Attitudes** |  |  |  |  |  |  |  |  |  |
| Organizational identification | -0.35 | 92.83 | 13 | 1903 | 0.37 | 0 | 0 | 2541.97 | 0.80 |
| Organizational commitment | -0.28 | 81.43 | 8 | 582 | -0.20 | 0 | 0 | 994.54 | 0.02 |
| Turnover intentions | 0.26 | 73.50 | 22 | 2641 | 1.48 | 3 | 0 | 0.01 | 3.12 |
| **Well-beings** |  |  |  |  |  |  |  |  |  |
| OBSE | -0.30 | 67.09 | 11 | 836 | 0.97 | 0 | 0 | 65.21 | 0.004 |
| Belongingness | -0.28 | 94.68 | 6 | 245 | -0.36 | 0 | 0 | 44994.56 | 1.27 |
| Job satisfaction | -0.34 | 74.09 | 12 | 1103 | 0.41 | 0 | 0 | 149.85 | 0.01 |
| Emotional exhaustion | 0.39 | 78.26 | 8 | 789 | 0.13 | 0 | 0 | 0.01 | 0.46 |
| **Behaviors** |  |  |  |  |  |  |  |  |  |
| Job performance | -0.28 | 72.47 | 17 | 1561 | -0.23 | 3 | 0 | 74.24 | 0.03 |
| OCB | -0.26 | 73.69 | 12 | 602 | 0.41 | 1 | 0 | 84.68^a^ | 0.07 |
| OCBO | -0.23 | 20.89 | 8 | 353 | -1.31 | 0 | 2 | 149.87 | 0 |
| OCBI | -0.24 | 37.84 | 10 | 475 | -0.43 | 0 | 0 | 0.01 | 0.04 |
| Organizational deviance | 0.59 | 97.09 | 23 | 20351 | -4.70 | 0 | 5 | 0.01 | 7.57^**^ |
| Interpersonal deviance | 0.66 | 97.27 | 21 | 14416 | -2.07 | 0 | 6 | 0.34 | 0.63 |

*Note.* The texts in italics are significant relationship from the meta-analysis results*.* ^a^ At least one of the p-value intervals contains no effect sizes, re-specifying the outpoints of 0.04<*p*<1.
