# Supplementary material for: Consequences of Workplace Ostracism: A Meta-Analytic Review
Source: Front Psychol. 2021 Aug 2;12:641302. doi: 10.3389/fpsyg.2021.641302 (PMC8365139; doi:10.3389/fpsyg.2021.641302)
Supplement: Supplementary file 1 [file Data_Sheet_1.ZIP › Supplementary Material Presentation./PRISMA 2009 flow diagram.doc]

**PRISMA 2009 Flow Diagram**

**Screening**

**Included**

**Eligibility**

**Identification**

Records identified through database searching
(n = 336)

Additional records identified through other sources
(n = 5)

Records after duplicates removed
(n = 318)

Records screened
(n = 97)

Records excluded
(n = 211)

Full-text articles assessed for eligibility
(n = 82)

Full-text articles excluded, with reasons
(n = 15)

Studies included in qualitative synthesis
(n = 3)

Studies included in quantitative synthesis (meta-analysis)
(n = 79)
